# Prognostic and predictive value of Phospho-p44/42 and pAKT in HER2-positive locally advanced breast cancer patients treated with anthracycline-based neoadjuvant chemotherapy

**DOI:** 10.1186/1477-7819-11-307

**Published:** 2013-11-30

**Authors:** Liang Huang, Tianwen Chen, Canming Chen, Sheng Chen, Yin Liu, Jiong Wu, Zhiming Shao

**Affiliations:** 1 Department of Breast Surgery, Fudan University Shanghai Cancer Center/Cancer Institute, 399 Ling-Ling Road, 200032 Shanghai, People’s Republic of China; 2 Department of Oncology, Shanghai Medical College, Fudan University, Shanghai, People’s Republic of China; 3 Department of Thyroid and Breast Surgery, Affiliated Nanshan Hospital of Guangdong Medical College, Shenzhen, People’s Republic of China

**Keywords:** Breast cancer, HER2/neu, Neoadjuvant chemotherapy, Overall survival, pAKT, Prognostic factors, Phospho-p44/42, Relapse-free survival

## Abstract

**Background:**

To evaluate the predictive and prognostic value of various molecular factors associated with the Ras/MAPK and PI3K/Akt signaling pathways in HER2-positive locally advanced breast cancer patients treated with anthracycline-based neoadjuvant chemotherapy (NAC).

**Methods:**

A total of 113 patients were recruited in this retrospective study. Core needle biopsies and excision samples were assessed through immunohistochemistry for various biomarkers, including IGF-1R, Phospho-p44/42, Ki67, pAKT, PTEN, p27, and cyclinD1. The changes in these biomarkers after NAC and their predictive and prognostic values were investigated.

**Results:**

Significant decreases in Ki67, Phospho-p44/42, and pAKT expression were observed after treatment (30.7% vs. 18.1%, 36.4% vs. 18.9%, and 35.1% vs. 16.4%, respectively). The decreases in Phospho-p44/42, pAKT, and Ki67 expression were strongly associated with the response to anthracycline treatment (*P* = 0.027, *P* = 0.031, and *P* = 0.008, respectively). In a multivariate survival analysis, Phospho-p44/42 expression after neoadjuvant chemotherapy and lymph node status were significant independent prognostic factors of both relapse-free survival and overall survival.

**Conclusions:**

Reductions in Ki-67, Phospho-p44/42, and pAKT expression are related to the clinical response to anthracycline-based NAC in HER2-positive breast cancer patients. High pAKT expression prior to NAC had a better clinical response. Phospho-p44/42 expression and lymph node status after NAC could be useful for determining relapse-free survival and overall survival.

## Background

Human epidermal growth factor receptor 2 (HER2) is a tyrosine kinase receptor; up to 25% of women with early breast cancer are HER2 positive. HER2 is associated with a more aggressive biological behavior, a higher likelihood of recurrence after initial treatment, and poorer prognosis [1]. Three publications have suggested that HER2 positivity is associated with a relative benefit from anthracycline-containing chemotherapy compared with non-anthracycline-containing regimens, which is in agreement with the results of two meta-analyses [2-6]. Since trastuzumab was approved for use in HER2-positive breast cancer patients, the prognosis of breast cancer patients has improved. When used as a single agent, overall response rates ranging from 15% to 30% have been reported [7].

Neoadjuvant chemotherapy (NAC) has been used in locally advanced breast cancer to convert previously unresectable cancer into operable cancer. More recently, it has been widely administered in primarily operable breast cancer to reduce tumor volume and allow conservative surgery. The complete pathological response rate for patients with HER2-positive tumors is nearly 23%, but the rate has been shown to increase to 40% with trastuzumab [8]. The additional use of anthracyclines in combination with trastuzumab is thought to explain the higher complete pathological response rates observed in the GeParQuinto trial [9].

HER2 overexpression may lead to increased receptor homodimerization and heterodimerization, which causes intrinsic receptor tyrosine kinase activity and induces phosphorylation of the intracellular domain. The growth factor receptors utilize several signaling pathways, including the Ras/MAPK pathway, which is important for mitogenic stimulation. They also activate the PI3K/Akt cascade, which has been shown to be important for cell survival and inhibiting apoptosis. The PI3K pathway is downstream of HER2 and is activated to catalyze the phosphorylation of inositol lipids to produce PIP3 from PIP2. PIP3 recruits protein kinases and activates the protein kinase B/AKT pathway. AKT phosphorylation can inhibit cell cycle arrest. The mitogen-activated protein kinase (MAPK) signaling pathway is known to be activated in breast cancer. Extracellular signal-related kinase (ERK), a member of the MAPK pathway, promotes cell proliferation, angiogenesis, cell differentiation, and cell survival.

Insulin-like growth factor receptor-1 (IGFR-1) is a transmembrane heterotetrameric protein. It is the dominant receptor for the IGF family of molecules, and it promotes the oncogenic transformation, growth and survival of cancer cells. IGF-I/II ligand binding induces intracellular tyrosine kinase activity and triggers a cascade of reactions involving signal transduction pathways, including the Ras, Raf, MAPK, and PI3K–AKT pathways. In breast cancer, IGFR-1 expression and activation have been linked to disease progression and poor prognosis [10,11].

To improve the efficacy of treatment in HER2-positive breast cancer patients, it is critical to study the correlation between HER2 signaling pathway activity and the efficacy of adjuvant treatment. Therefore, we initiated a retrospective study to collect serial samples of HER2-positive breast cancer for molecular analyses in patients undergoing anthracycline-based neoadjuvant chemotherapy.

## Methods

### Ethics statement

The retrospective study was approved by the Ethics Committee of Shanghai Cancer Center. Written informed consent was obtained from each patient involved in the study.

### Patients and clinical samples

From May 2002 to September 2007, 113 patients with HER2-overexpressing (defined as either 3+ or 2+ with confirmed c-erbB2 gene amplification by fluorescence *in situ* hybridization) stage II to III breast cancer were retrospectively recruited [12]. Core needle biopsy was performed for every patient to confirm the diagnosis of invasive cancer. A complete history, including patient characteristics, clinical and imaging examinations, and pathologic assessments of the morphologic and biologic features of the cancers, was collected. Patients with metastatic diseases, inflammatory breast cancer or male breast cancer were not included in this study. All patients were treated with CEF (cyclophosphamide 600 mg/m^2^, epirubicin 80 mg/m^2^ and fluorouracil 500 mg/m^2^, q3w) or NE (vinorelbine 25 mg/m^2^ on days 1 and 8 and epirubicin 60 mg/m^2^ on day 1, q3w). Following completion of neoadjuvant treatment, all patients underwent breast surgery. For adjuvant chemotherapy, 74.3% of cases had an anthracycline-based regimen, and 10.6% of cases had a paclitaxel regimen; two patients received trastuzumab treatment for 1 year. Other standard therapies, including radiation therapy and endocrine therapy, were administered at the discretion of the treating clinician following NCCN guidelines. All patients were followed-up every 3 months for the first year and then every 6 months until death.

### Assessment of the response to neoadjuvant chemotherapy

All surgical specimens were submitted for pathological evaluation. A complete pathological response was defined as no residual invasive carcinoma in the breast or lymph nodes. The clinical stage and size of the primary tumor measured by MRI or ultrasonography were recorded before treatment. The primary tumor was measured as the product of its greatest diameter. The clinical response was evaluated at each cycle of chemotherapy and prior to definitive surgery on day 21 of the last cycle of chemotherapy as the product of the primary tumor diameters and the axillary clinical status and classified as a complete response, partial response, stable disease, or progressive disease according to the solid tumors criteria (RECIST 1.1).

### Immunochemistry

Immunohistochemistry was performed on deparaffinized sections of all core needle biopsies and surgical tumor samples. Antibodies for P27, Phospho-p44/42 and pAKT required antigen retrieval in a pressure cooker. Briefly, after antigen retrieval, endogenous peroxidases and biotin were blocked with 3% hydrogen peroxide and an Avidin-Biotin Blocking Kit (Vector, CA), respectively. This was followed by incubation with the primary antibody for 60 minutes at room temperature, the appropriate secondary antibody (Dako), labeled streptavidin-horseradish-peroxidase (Dako), DAB chromogen, and 0.2% osmium tetroxide (Sigma Chemicals, St Louis, MO), followed by counterstaining with light hematoxylin. Appropriate positive controls for each antibody and negative controls using species-matched immunoglobulin to replace the primary antibody were run with each batch. Positive tumor cells were quantified by evaluating at least 1,000 cells and expressed as percentages. Samples were evaluated by two trained pathologists (Dr. Zou, Dr. Li) who were blinded to the patient background and clinical outcome. If the difference between the two results was more than 10%, a third pathologist (Dr. Zhou) was consulted.

The cut-off for estrogen receptor (clone 1D5, Dako) and progesterone receptor (clone PgR 636, Dako) positivity was 1% of tumor cells with positive nuclear staining. For Phospho-p44/42 (clone 20G11, Cell Signaling) and pAKT (clone 736E11, Cell Signaling), the cut-off for positive expression was 20% of cells with nuclear and cytoplasmic expression [13]. The cut-off for Ki67 (clone MIB-1, Dako) positivity was 14% of tumor cells with positive nuclear staining. The cut-off for other markers was 10%, including nuclear staining for P27 (clone SX53G8, Dako) and cyclinD1 (clone EP12, Dako), membranous and cytoplasmic staining for IGF-1R (clone 3027, Cell Signaling), and cytoplasmic and nuclear staining for PTEN (clone 6H2.1, Dako) [14-16]. The reduction between the pre-NAC average percentage and post-NAC average percentage was defined as the reduction cut-off for Ki67, pAKT, and Phospho-p44/42 (13%, 19%, and 18%, respectively).

### Statistical analysis

Descriptive statistics were calculated to summarize patient characteristics, tumor size, and the biomarker levels in the core needle biopsies and surgical tumor samples. Biomarker expression levels in pre- and post-chemotherapy tumor samples were compared using a paired *t* test. The initial biomarker levels were compared between responders and non-responders using the chi-square test. Fisher’s exact test was performed when necessary. A simultaneous analysis of the biomarkers that were significantly predictive of tumor response in the univariate analysis was performed using a multivariate logistic regression.

Survival results were last updated in September 2012. Relapse-free survival was defined as the elapsed time between the date of first diagnosis and the date of first relapse. Overall survival was calculated from the date of diagnosis to the date of death or last follow-up. Patients without events or death were censored at the last follow-up. Survival curves were established according to the Kaplan-Meier method. The log-rank test was used for univariate comparison of survival endpoints. A Cox regression was used to assess the relative influence of prognostic factors on relapse-free survival and overall survival. All tests were considered significant at a two-sided *P* < 0.05. All analyses were performed using SPSS 17.0 (SPSS, Chicago, IL).

## Results

### Clinical characteristics and responses to NAC

A total of 113 HER2-positive breast cancer patients were recruited in this retrospective study. The average age of patients at diagnosis was 49 (range 26 to 78) years; 69 patients were premenopausal at presentation. There were 67 patients with a baseline tumor size greater than 5 cm. The CEF regimen was given to 55 patients, and the other patients received the NE regimen. The mean number of NAC cycles was 3.52 (range 1 to 8), and the objective response (complete response + partial response) and non-response rates (progressive disease + stable disease) were 70.8% and 29.2%, respectively. Eight patients underwent breast-conserving surgery. The clinical characteristics of these patients are shown in Table 1.

**Table 1:** Clinical characteristics of HER2-positive breast cancer patients and the univariate analysis of predictive biomarkers of the response to anthracyclines

**Factors**	**Number of patients (%)**	**Clinical response**			**Pathological response**		
		**Stable disease + progressive disease**	**Partial response + complete response**	***P***	**Complete pathological response**	**Incomplete pathological response**	***P***
Age							
45 years	34 (30%)	10	24	0.975	3	31	0.450
≥45 years	79 (70%)	23	56		11	68	
Menopausal status							
Postmenopausal	69 (61%)	20	49	0.949	7	62	0.364
Premenopausal	44 (39%)	13	31		7	37	
Regimen							
CEF	55 (49%)	15	40	0.660	9	46	0.212
NE	58 (51%)	18	40		5	53	
Tumor size							
≤5 cm	46 (41%)	21	25	0.001	4	42	0.323
>5 cm	67 (59%)	12	55		10	57	
Lymph node status							
0	43 (38%)	-	-	-	-	-	-
1 to 3	31 (27%)	-	-	-	-	-	-
4 to 9	25 (22%)	-	-	-	-	-	-
≥10	14 (13%)	-	-	-	-	-	-
Pre-estrogen receptor							
Negative	78 (69%)	21	57	0.426	10	68	0.835
Positive	35 (31%)	12	23		4	31	
Pre-progesterone receptor							
Negative	83 (73%)	24	59	0.911	9	74	0.518
Positive	30 (27%)	9	21		5	25	
Pre-pMAPK							
Negative	34 (30%)	13	21	0.166	8	26	0.855
Positive	79 (70%)	20	59		6	73	
Pre-pAKT							
Negative	30 (27%)	14	16	0.014	4	26	0.855
Positive	83 (73%)	19	64		10	73	
Pre-PTEN							
Negative	40 (35%)	13	27	0.568	5	35	0.979
Positive	73 (65%)	20	53		9	64	
Pre-P27							
Negative	35 (31%)	15	20	0.432	5	40	0.737
Positive	68 (69%)	18	50		9	59	
Pre-IGF-1R							
Negative	47 (42%)	12	35	0.469	5	42	0.634
Positive	66 (58%)	21	45		9	57	
Pre-cyclinD1							
Negative	42 (37%)	14	28	0.458	4	38	0.477
Positive	71 (63%)	19	52		10	61	
Pre-Ki67							
Negative	43 (38%)	15	28	0.298	2	41	0.076
Positive	70 (62%)	18	52		12	58	

IGF-1R, insulin-like growth factor 1 receptor; pAKT, phosphorylated AkT; pMAPK, phosphorylated mitogen-activated protein kinase.

### Changes in biomarker expression after NAC

**Figure 1:**
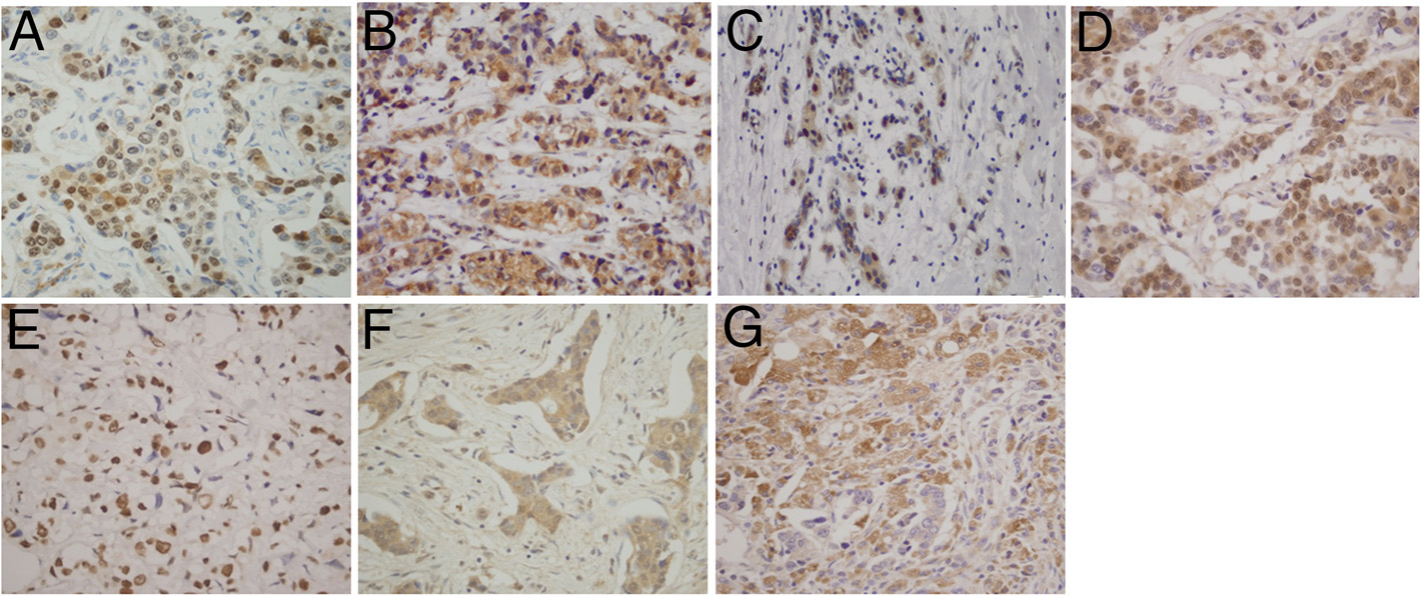
Positive immunohistochemical expression: (A) Ki67, (B) Phospho-p44/42, (C) pAKT, (D) p27, (E) PTEN, (F) IGF-1R, (G) cyclinD1.

**Figure 2:**
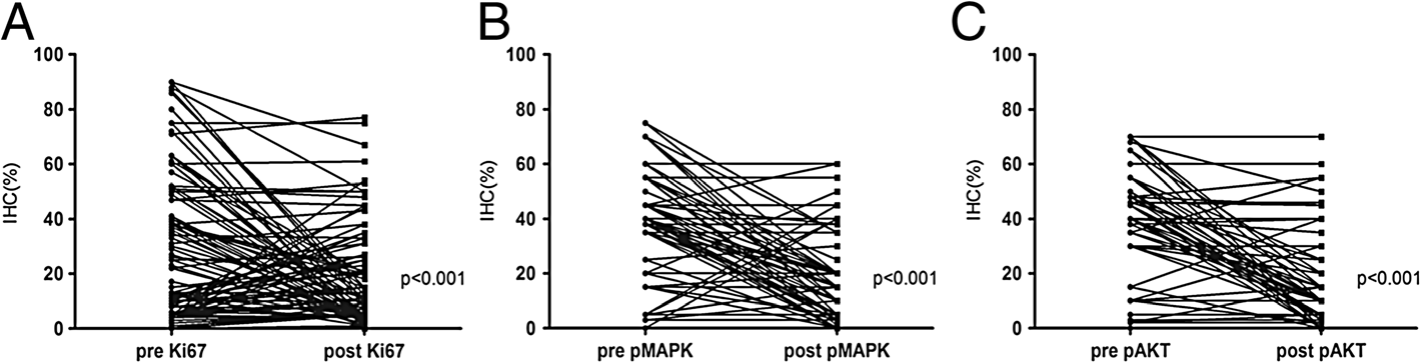
**Ki67, Phospho-p44/42 and pAKT expression were significantly decreased after anthracycline treatment. (A)** Ki67, **(B)** Phospho-p44/42, **(C)** pAKT.

The expressions of various biomarkers (in Figure 1) before and after NAC were compared in 99 patients who did not achieve complete pathological response after NAC. A paired *t* test analysis found no significant changes in p27, PTEN, IGF-1R, or cyclinD1 expression (15.7% vs. 16.5%, 17.0% vs. 18.3%, 49.0% vs. 51.0%, and 18.2% vs. 16.9%, respectively). A significant decrease in Ki67, Phospho-p44/42, and pAKT expression was observed after treatment (30.7% vs. 18.1%, 36.4% vs. 18.9%, and 35.1% vs. 16.4%, respectively). We defined positive-staining tumor cells decreasing by more than the cut-off value as positive decrease in biomarker expression. The details are shown in Table 2. Ki67, Phospho-p44/42, and pAKT expression were all significantly decreased after anthracycline-based neoadjuvant chemotherapy, as shown in Figure 2.

**Table 2:** Univariate analysis of relapse-free survival and overall survival

**Factor**	**Relapse-free survival**		**Overall survival**		**Factor**	**Relapse-free survival**		**Overall survival**	
	***P***	**Hazard ratio (95% confidence interval)**	***P***	**Hazard ratio (95% confidence interval)**		***P***	**Hazard ratio (95% confidence interval)**	***P***	**Hazard ratio (95% confidence interval)**
Age									
<45y vs. ≥45y	0.724	1.2 (0.5 to 2.7)	0.256	1.0 (0.9 to 1.0)					
Menopausal status									
Premenopausal vs. Postmenopausal	0.663	0.9 (0.5 to 1.5)	0.822	1.0 (0.6 to 2.0)					
Regimen									
CEF vs. NE	0.479	1.2 (0.7 to 2.0)	0.568	1.2 (0.6 to 2.2)					
Tumor size									
≤5 cm vs. >5 cm	0.784	1.1 (0.6 to 2.0)	0.456	1.3 (0.6 to 2.8)					
Lymph node status									
Negative									
1 to 3 nodes +	0.007	2.7 (1.3 to 5.6)	0.012	4.3 (1.4 to 13.3)					
4 to 9 nodes +	<0.001	4.6 (2.2 to 9.5)	0.001	7.2 (2.4 to 21.9)					
≥10 nodes +	<0.001	6.8 (3.0 to 15.9)	<0.001	13.7 (4.3 to 44.4)					
Pre-estrogen receptor					Post-estrogen receptor				
Negative vs. positive	0.252	1.4 (0.8 to 2.3)	0.620	1.2 (0.6 to 2.2)	Negative vs. positive	1.000	1.0 (0.5 to 1.8)	0.540	0.8 (0.4 to 1.7)
Pre-progesterone receptor					Post-progesterone receptor				
Negative vs. positive	0.724	0.9 (0.5 to 1.6)	0.334	0.7 (0.3 to 1.5)	Negative vs. positive	0.841	1.0 (0.6 to 2.0)	0.754	0.9 (0.4 to 2.0)
Pre-Ki67					Post-Ki67				
Negative vs. positive	0.840	1.1 (0.6 to 1.8)	0.647	0.9 (0.5 to 1.6)	Negative vs. positive	0.260	1.4 (0.8 to 2.3)	0.434	1.3 (0.7 to 2.4)
Pre-pMAPK					Post-pMAPK				
Negative vs. positive	0.174	0.7 (0.4 to 1.2)	0.788	1.1 (0.6 to 2.2)	Negative vs. positive	0.019	2.0 (1.1 to 3.5)	<0.001	3.7 (1.9 to 7.0)
Pre-pAKT					Post-pAKT				
Negative vs. positive	0.224	0.7 (0.4 to 1.2)	0.104	0.6 (0.3 to 1.1)	Negative vs. positive	0.551	0.8 (0.5 to 1.5)	0.548	0.8 (0.4 to 1.7)
Pre-PTEN					Post-PTEN				
Negative vs. positive	0.450	0.8 (0.5 to 1.4)	0.538	0.8 (0.4 to 1.5)	Negative vs. positive	0.622	0.9 (0.5 to 1.6)	0.571	1.3 (0.6 to 2.7)
Pre-P27					Post-P27				
Negative vs. positive	0.929	1.0 (0.6 to 1.6)	0.439	0.8 (0.4 to 1.5)	Negative vs. positive	0.220	1.4 (0.8 to 2.5)	0.317	1.4 (0.7 to 2.9)
Pre-IGF-1R					Post-IGF-1R				
Negative vs. positive	0.728	1.1 (0.7 to 1.8)	0.874	1.1 (0.6 to 2.0)	Negative vs. positive	0.388	0.8 (0.5 to 1.3)	0.760	1.1 (0.6 to 2.2)
Pre-cyclinD1					Post-cyclinD1				
Negative vs. positive	0.606	1.2 (0.7 to 2.0)	0.814	1.1 (0.6 to 2.1)	Negative vs. positive	0.602	1.2 (0.7 to 2.1)	0.912	1.0 (0.5 to 2.1)

IGF-1R, insulin-like growth factor 1 receptor; pAKT, phosphorylated AKT; pMAPK, phosphorylated mitogen-activated protein kinase.

### Predictors and response to NAC

**Figure 3:**
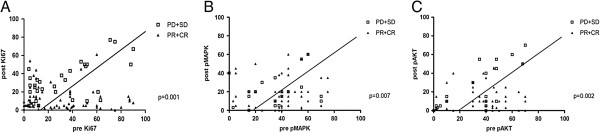
**Correlation between pretreatment and post-treatment markers and the pathological response.** Values below the line indicate the percentage decrease compared to the cut-off value. **(A)** Ki67, **(B)** Phospho-p44/42, **(C)** pAKT.

Biomarkers and clinical characteristics were examined to investigate their value in predicting the NAC response (Table 2). Univariate analysis demonstrated that tumor size and pre-pAKT were predictive factors of the response to anthracyclines (*P* = 0.001 and *P* = 0.014, respectively). Multivariate analysis demonstrated that primary tumor size and pAKT expression remained independent predictive factors of the clinical response to anthracycline-based NAC (*P* = 0.012 and *P* = 0.031, respectively). However, no biomarker could predict a pathologic complete response. We also found that the clinical response was coincident with decreased biomarker expression, including Ki67, pAKT, and Phospho-p44/42 (*P* = 0.001, *P* = 0.002, and *P* = 0.007, respectively), as shown in Figure 3.

### Prognostic markers

**Figure 4:**
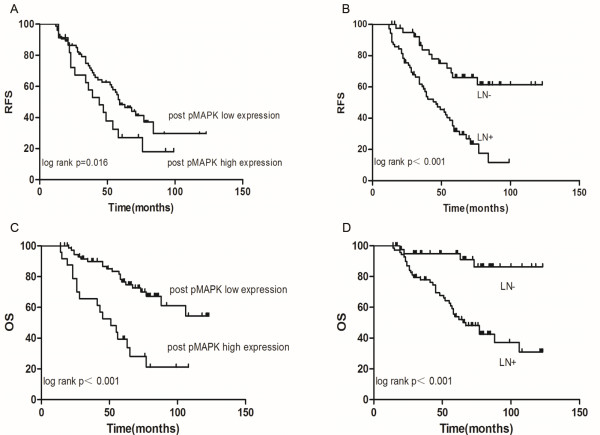
**Kaplan-Meier curves.** Curves for relapse-free survival according to **(A)** post-Phospho-p44/42 and **(B)** lymph node status. Curves for overall survival according to **(C)** post-Phospho-p44/42 and **(D)** lymph node status.

The median follow-up time was 60 months (ranging from 14 to 123 months). The overall 5-year relapse-free survival was 50.4%, and the overall survival was 72.6%. A univariate analysis (Table 2) demonstrated that the number of positive lymph nodes and post-Phospho-p44/42 expression were prognostic factors for relapse-free survival. In the multivariate analysis, the number of positive lymph nodes (hazard ratio, 2.0; 95% confidence interval, 1.6 to 2.6; *P* < 0.001) and post-Phospho-p44/42 expression (hazard ratio, 2.3; 95% confidence interval 1.3 to 4.1; *P* < 0.001) remained significantly independent prognostic factors. In the univariate analysis (Table 2), lymph node status (*P* < 0.001) and post-Phospho-p44/42 expression (*P* = 0.019) showed clear associations with overall survival. In the multivariate analysis, lymph node status (hazard ratio, 2.3; 95% confidence interval, 1.7 to 3.3; *P* < 0.001) and post-Phospho-p44/42 expression (hazard ratio, 4.3; 95% confidence interval, 2.2 to 8.4; *P* < 0.001) were also significant predictors of overall survival. Representative survival curves are shown in Figure 4.

## Discussion

To our knowledge, this is the largest analysis of PI3K-Akt and MAPK pathway activation in HER2-overexpressing breast cancer patients who received an anthracycline-based neoadjuvant chemotherapy regimen without trastuzumab.

Owing to its aggressive nature and poor prognosis, a number of preclinical and clinical studies have focused on the HER2-positive subtype. The use of anthracyclines in neoadjuvant treatments for HER2-positive breast cancer in addition to trastuzumab is still controversial [17].

HER2 signaling activates pathways (PI3K/Akt and Ras/MAPK) regulating cell cycle progression and cell proliferation. In HER2-overexpressing MBC group, Gori (*et al*. found that Phospho-p44/42 and pAKT were not associated with the clinical outcome, although low Phospho-p44/42 expression showed a trend for association with a longer overall survival [18]. In our study, the pre-pAKT and pre-Phospho-p44/42 expression rates were high, and this finding has been confirmed by other recent studies [19-21]. The results indicate that the Ras/MAPK and PI3K/Akt pathways are universally active in locally advanced breast cancer with HER2 overexpression. However, the PI3K/Akt and Ras/MAPK pathways have been related to resistance to doxorubicin and paclitaxel in breast cancer cells [22,23]. The level of pre-pAKT expression has been shown to have a significant correlation with the objective response rates to anthracycline treatment. It is possible that Akt isoforms have a distinct impact on the cellular resistance to a given drug and, in fact, Akt activity does not confer equal resistance to different chemotherapeutic agents. For example, the overexpression of constitutively active Akt isoforms in HeLa cells has been shown to induce isoform-specific sensitivity to doxorubicin [24]. The role of pAKT in the neoadjuvant setting is still controversial, owing to limited investigations, even in large clinical trials. However, decreases in Phospho-p44/42 and pAKT expression are related to the response to anthracyclines. Higher levels of active MAPK may have aggressive biological behavior, which has been found to be associated with lymph node metastasis [25]. In the TNBC subgroup, high ERK protein expression levels and shorter survival times have been observed [26]. After anthracycline-based adjuvant treatment, a higher score was significantly associated with poorer survival following relapse compared to a lower expression score among patients with MAPK overexpression [21].

Furthermore, few studies have presented precise values for pAKT and Phospho-p44/42 in neoadjuvant chemotherapy. In our study, low Phospho-p44/42 expression after neoadjuvant chemotherapy was a strong prognostic factor for these patients. Patients with high post-Phospho-p44/42 expression had a higher recurrence rate (up to 68%) in the first 5 years, while patients with low post-Phospho-p44/42 expression had a lower recurrence rate (45%), and the survival difference between the two groups was highly significant. It is possible that decreased pAKT and Phospho-p44/42 expression promotes tumor apoptosis and inhibits tumor proliferation, resulting in a survival benefit for HER2-positive breast cancer patients treated with anthracyclines. It is also possible that topoisomerase IIα expression is regulated by Ras pathways and tumor proliferation status. The activation of the Ras/Raf/MAPK pathway has been shown to be involved in the induction of MRP-1 activity and topoisomerase IIα downregulation, which are the main mechanisms of anthracycline resistance [27].

Higher levels of the proliferation marker Ki67 are associated with poorer survival in breast cancer patients, but we found no prognostic value for pre-NAC Ki67, post-NAC Ki67, or the Ki67 fold change. Other studies have reached controversial conclusions, and it is therefore difficult to choose reasonable predictive and prognostic factors among pre-NAC Ki67, post-NAC Ki67, and Ki67 reduction [14,28-30].

This study has limitations common to all retrospective analyses, and it lacked a control group, such as patients treated with trastuzumab-containing NAC. However, based on results of big clinical trials, such as HERA, BCIRG 006, NCCTG N9831, and NSABP B-31, trastuzumab was approved for adjuvant chemotherapy after 2005. In our study, 71% patients were treated before 2005 when most patients did not receive trastuzumab in developing countries. Additionally, the number of patients was small. However, the scientific and clinical community must establish and evaluate these biomarkers and standardize cut-off levels. Although a measurement of topoisomerase II-α amplification was not within the scope of this study, a possible explanation for our findings may be that other confounding molecular factors are involved in the mechanism of the anthracycline response in HER2-positive patients.

## Conclusions

In HER2-positive breast cancer patients treated with anthracyclines, the expression of pAKT, Phospho-p44/42, and Ki67 decreased significantly after treatment. Furthermore, patients with high pAKT expression before NAC had higher objective response rates to anthracyclines. The results of our study demonstrate that Phospho-p44/42 expression after neoadjuvant chemotherapy is a strong predictor of outcome. It will be necessary and valuable to further evaluate potential therapeutic targets of the PI3K and MEK signaling pathways in HER2-positive breast cancer patients.

## Abbreviations

ERK: Extracellular signal-related kinase; HER2: Human epidermal growth factor Receptor 2; IGF-1R: Insulin-like growth factor 1 receptor; MAPK: Mitogen-activated protein kinase; NAC: Neoadjuvant chemotherapy; pAKT: Phospho-protein kinase B; Phospho-p44/42: Phospho-mitogen-activated protein kinase; pMAPK: Phosphorylated mitogen-activated protein kinase.

## Competing interests

All authors declare that they have no potential conflict of interest.

## Authors’ contributions

LH and T-WC have made substantial contributions to the conception and design of the study, and the acquisition of data. SC and YL analyzed and interpreted the data. JW and Z-MS revised the manuscript critically for important intellectual content. C-MC gave final approval of the version to be published. All authors read and approved the final manuscript.
